# Risk factors for maternal pyrexia, infection and sepsis in four hospitals providing maternity care in New South Wales, Australia: a cohort study

**DOI:** 10.3389/fgwh.2025.1532500

**Published:** 2025-09-26

**Authors:** Kelly Thompson, Shuyao Yan, Gary Low, Amanda Henry

**Affiliations:** 1 Global Women’s Health Program, The George Institute for Global Health, UNSW Medicine and Health, Sydney, NSW, Australia; 2 School of Population Health, Faculty of Medicine, UNSW, Sydney, NSW, Australia; 3 Sydney Nursing School, University of Sydney, Sydney, NSW, Australia; 4 Nepean Blue Mountains Local Health District, Kingswood, NSW, Australia; 5 Central Coast Local Health District, Gosford, NSW, Australia; 6 Sydney Medical School, University of Sydney, Camperdown. NSW, Australia; 7 Discipline of Women’s Health, School of Clinical Medicine, UNSW, Sydney, NSW, Australia; 8 St George Hospital, South East Sydney Local Health District, Kogarah, NSW, Australia

**Keywords:** maternal sepsis, maternal infection, maternal, sepsis, infection, risk factors, antimicrobial resistance

## Abstract

**Introduction:**

Maternal sepsis is a leading cause of maternal mortality. In Australia, it is the third most common cause of maternal death despite a low overall maternal death rate (5.8 per 100,000 births). The objective of this study was to examine risk factors for maternal pyrexia, infection and sepsis, adherence to WHO maternal antibiotic prophylaxis guidelines and resistance patterns in women with Group B Streptococcus (GBS).

**Methods:**

We conducted a retrospective observational cohort study using routinely collected data from four hospitals providing maternity care in New South Wales, Australia, including pathology data from one hospital. Women who gave birth between January 1, 2018, and December 31, 2020, were included. Definitions for pyrexia, infection, and sepsis were based on medical notes and the obstetrically modified Sequential Organ Failure Assessment criteria. We used multivariable logistic regression to identify risk factors and descriptive statistics to evaluate antibiotic prophylaxis adherence and resistance.

**Results:**

Out of 23,016 women, 2,650 (11.5%) experienced pyrexia, infection, or sepsis. Women with pyrexia, infection, or sepsis were more likely to report a history of substance use and less likely to receive influenza vaccination. Hospital-based (non-continuity) midwifery care, nulliparity, and emergency Caesarean section and instrumental vaginal birth were associated with increased risk of pyrexia, infection, or sepsis. Documented adherence to antibiotic prophylaxis guidelines was suboptimal, with 35% of Caesarean sections and 29% of severe perineal tear cases documented as receiving antibiotics. In the subset of women with available pathology data, GBS screening was performed in 72.6% of cases, with 19.4% testing positive. Resistance to erythromycin (33.4%) and clindamycin (30.9%) was high, though no resistance to penicillin or ampicillin was observed.

**Conclusion:**

Demographic and labour/birth factors conveying an increased risk of pyrexia, infection or sepsis were broadly in line with previous studies. Adherence to WHO prophylaxis guidelines was poorly documented and increased rates of antibiotic resistance to erythromycin and clindamycin were observed. Ongoing monitoring of resistance patterns and improving guideline adherence is important to optimise care.

## Introduction

Maternal sepsis is life-threatening organ dysfunction caused by infection during pregnancy, childbirth, post-abortion, or the postpartum period (1). It is the third leading cause of maternal death globally, responsible for 10.7% of maternal deaths. In Australia, although the overall maternal death rate is low (5.8 per 100,000 births), sepsis remains a leading cause of maternal mortality (2).

Risk factors for maternal sepsis include demographic characteristics, pregnancy conditions, and birth-related factors. Key demographic factors are older maternal age, pre-existing cardiovascular disease, impaired glucose tolerance, minority status, and obesity (3, 4). Pregnancy-related risks include multiple gestation, high parity, and Caesarean section (CS), with emergency CS posing a higher risk than elective CS (4, 5). Other contributing factors are operative vaginal delivery, preterm birth, preterm premature rupture of membranes (PPROM), retained products of conception, and postpartum haemorrhage (4, 6).

There are limited Australian data on risk factors for maternal pyrexia, infection, and sepsis. In addition, data on optimal use of antibiotic prophylaxis in accordance with WHO guidelines for maternal antibiotic prophylaxis in the peripartum period (7) are lacking and there are increasing concerns about antimicrobial resistance in maternity populations (8–10).

Our objective was to identify risk factors for maternal pyrexia, infection, and sepsis in four tertiary and regional hospitals in New South Wales (NSW), Australia. We hypothesised that these factors would align with those identified in other high-income countries (3, 4, 11–15). Additionally, we examined current antimicrobial use in the peripartum period and assessed antibiotic resistance in women with Group B Streptococcus (GBS).

## Materials and methods

### Study design and setting

We conducted a retrospective observational cohort study using routinely collected maternity data from two metropolitan and two regional/rural hospitals in New South Wales (NSW). Additionally, we analysed pathology data from a subset of participants at one tertiary hospital. Ethics approval was granted by the Sydney Children's Hospitals Network Human Research Ethics Committee (Application ID: 2020/ETH02817).

### Participant recruitment

We included all adult women (aged 18 years and older) who gave birth at St George Hospital (SGH), Nepean Hospital, Blue Mountains Hospital, or Lithgow Hospital, NSW, between January 1, 2018, and December 31, 2020. Participants were selected based on the availability of routinely collected maternity data.

### Definitions of pyrexia, infection, and sepsis

Pyrexia was defined as a fever, pyrexia or non-specific fever recorded in the medical record, generally in Australia this would be a temperature of ≥38 °C. Infection was identified if a woman had an identifiable cause of infection or had pyrexia with a suspected infection. Sepsis was defined as infection accompanied by organ failure according to the obstetrically modified Sequential Organ Failure Assessment (omSOFA) criteria (16) or sepsis documented in the medical record.

### Outcomes

The primary outcome was to identify risk factors associated with maternal pyrexia, infection, and sepsis. Secondary outcomes were analysed in a subset of participants from SGH with available pathology data and included antibiotic prophylaxis in alignment with WHO recommendations (7) (CS, GBS infection, manual removal of placenta, PPROM, 3rd or 4th degree perineal tear), as well as a description of antibiotic resistance profiles in women with GBS. We anticipated that antimicrobial prophylaxis would comply with guidelines in over 80% of GBS cases and predicted antibiotic resistance to be less than 1% for benzylpenicillin and over 10% for clindamycin among GBS positive women.

### Data collection and extraction

We extracted routine antenatal, intrapartum, and immediate postnatal data from the eMaternity database across all sites, covering live births and stillbirths of at least 20 weeks' gestation and 400 g birth weight. This database is highly accurate for standard data fields, though less so for certain obstetric diagnoses (17, 18). Collected variables included demographics, admission details, past medical and medication history, labour and delivery details.

For SGH, we also accessed maternal urine, blood, and wound culture results from the Auslab Pathology database, and analysed laboratory data. Data from eMaternity and Auslab were linked using probabilistic matching of medical record numbers and dates of birth, with the final dataset anonymised and stored at The George Institute for Global Health.

### Statistical analysis

We describe the data using descriptive statistics and report parametric data as means ± standard deviations and non-parametric data as medians and interquartile ranges. We initially used a generalised linear mixed-effects model (glmer) to account for hospital and multiple admissions effects but found minimal impact. Subsequently, we used a logistic regression model [generalised linear models (glm)] excluding these variables. Antibiotic administration was excluded from the multivariable analysis as it is generally considered to be more of a consequence of sepsis or infection, rather than a cause. We analysed the presence or absence of pyrexia, infection, or sepsis. Due to multicollinearity between “labour intervention” and “genital tract trauma,” separate models were used by way of sensitivity analysis. A further sensitivity analysis addressed missing values in CS cases by replacing them with either elective or emergency CS. The “Emergency CS model” and “Genital Tract Trauma model” results were combined to calculate the odds ratio (OR). In the pathology subset, differences were assessed using Student's *t*-test, Mann–Whitney *U* test, and chi-square or Fisher exact tests as appropriate. Data were cleaned and prepared in Microsoft Excel, then analysed using R (Version 4.4.0). Laboratory data were analysed using SPSS (IBM SPSS Statistics v26).

## Results

During the study period, 23,016 women gave birth. Of these, 2,650 (11.5%) experienced pyrexia, infection, or sepsis. The demographic characteristics of the overall cohort are provided in the Supplementary Table S1. The average maternal age was 30.3 years, with over half of the women classified as overweight or obese (BMI ≥ 25). Most women were married (74.4%), and many (37.0%) were born overseas. Women with pyrexia, infection, or sepsis had significantly higher rates of substance use and lower rates of influenza vaccination compared to those without (Table 1).

**Table 1:** Demographic, pregnancy and birth characteristics.

Characteristics	No pyrexia/infection/sepsis *n* = 20,366	Pyrexia/infection/sepsis *n* = 2,650	Odds ratio (95%CI)^a^
Demographic characteristics			
Age in years (Mean ± SD)	30.5 ± 5.4	30.2 ± 5.4	0.99 (0.98–1)
Body Mass Index categories (*n* %)			
Underweight (<18.5)	841 (4.1)	110 (4.2)	1.13 (0.89–1.43)
Normal (18.5–24.9) (ref.)	9,337 (45.8)	1,070 (40.4)	
Overweight (25.0–29.9)	5,087 (25)	735 (27.7)	1.18 (1.06–1.32)
Obese (>30.0)	5,101 (25)	735 (27.7)	1.08 (0.96–1.21)
Born in Australia (*n* %)	12,383 (60.8)	1,726 (65.1)	1.09 (0.98–1.21)
Marital status (*n* %)			
Married/De-facto (ref.)	15,413 (75.8)	1,935 (73.1)	
Never married	4,399 (21.6)	613 (23.1)	1 (0.89–1.13)
Divorced	115 (0.6)	17 (0.6)	1.03 (0.54–1.83)
Other	402 (2)	83 (3.1)	1.18 (0.82–1.66)
History of endocrine disease (*n* %)	3,152 (15.5)	463 (17.6)	1.09 (0.96–1.23)
History of surgery (*n* %)	5,643 (27.8)	735 (27.9)	0.98 (0.88–1.09)
Substance use (*n* %)	240 (1.2)	53 (2)	1.54 (1.05–2.23)
Alcohol use (*n* %)	579 (2.9)	73 (2.8)	0.98 (0.73–1.3)
Smoking (*n* %)	2,481 (12.3)	361 (13.7)	0.93 (0.8–1.08)
History of influenza vaccination (*n* %)	5,757 (28.3)	661 (24.9)	0.84 (0.76–0.94)
History of pertussis vaccination (*n* %)	8,925 (43.8)	1,025 (38.7)	0.92 (0.83–1.01)
Type of hospital (*n* %)			
Regional	227 (1.1)	48 (1.8)	—
Rural	73 (0.4)	14 (0.5)	—
Tertiary	20,065 (98.5)	2,588 (97.7)	—
Pregnancy characteristics			
Gestation when first antenatal visit (Mean ± SD)	17 ± 6.3	17.6 ± 5.9	1 (0.99–1.01)
Total antenatal visits (Mean ± SD)	8.2 ± 3	7.9 ± 3	—
Model of antenatal care (*n* %)			
Hospital-based Medical^b^ (ref.)	1,064 (5.2)	83 (3.1)	—
Hospital-based Midwifery	16,686 (81.9)	2,410 (90.9)	1.97 (1.54–2.56)
Hospital high-risk maternity care	303 (1.5)	16 (0.6)	0.7 (0.38–1.22)
Midwifery Caseload/Midwifery Team	1,319 (6.5)	70 (2.6)	1.1 (0.77–1.57)
Private	505 (2.5)	29 (1.1)	0.51 (0.22–1.02)
Shared Care	477 (2.3)	41 (1.5)	1.47 (0.97–2.22)
No Antenatal Care	12 (0.1)	1 (0)	9.78 (0.4–132.05)
Parity (*n* %)			
0 (ref.)	7,959 (39.1)	1,414 (53.4)	
1	6,931 (34)	687 (25.9)	0.71 (0.64–0.8)
2	3,229 (15.9)	316 (11.9)	0.66 (0.57–0.77)
3	1,285 (6.3)	128 (4.8)	0.68 (0.54–0.85)
4+	953 (4.7)	103 (3.9)	0.73 (0.56–0.94)
Multiple pregnancy (*n* %)	624 (3.1)	180 (6.8)	1.02 (0.81–1.27)
History of mental health disorders (*n* %)	5,332 (26.3)	750 (28.4)	0.97 (0.87–1.08)
Maternal hypertension (*n* %)	1,324 (6.5)	216 (8.2)	1 (0.82–1.21)
Diabetes (*n* %)	3,666 (18)	502 (18.9)	0.89 (0.79–1.01)
Threatened premature labour^c^ (*n* %)	562 (2.8)	238 (9)	1.82 (1.46–2.27)
PPROM (*n* %)	523 (2.6)	317 (12)	4.58 (3.77–5.56)
Antepartum haemorrhage (*n* %)	642 (3.2)	163 (6.2)	1.24 (0.98–1.55)
Birth characteristics			
Mode of Delivery (*n* %)			
Caesarian section Elective Caesarean section	2,668 (13.1)	337 (12.7)	2.77 (2.27–3.37)
Emergency Caesarean section	2,306 (11.3)	729 (27.5)	5.32 (4.53–6.26)
Unknown indication for Caesarean section	1,881 (9.2)	576 (21.7)	—
Instrumental			
Instrumental with Induction of Labour	1,334 (6.6)	210 (7.9)	2.88 (2.33–3.56)
Spontaneous Instrumental	978 (4.8)	125 (4.7)	2.33 (1.82–2.97)
Normal Vaginal DeliveryVaginal birth with Induction of Labour	5,952 (29.2)	390 (14.7)	1.23 (1.04–1.47)
Spontaneous vaginal birth (ref.)	5,160 (25.3)	249 (9.4)	—
Vaginal breech with Induction of Labour	51 (0.3)	22 (0.8)	4.07 (1.84–8.55)
Spontaneous Vaginal breech	36 (0.2)	12 (0.5)	3.61 (1.3–8.97)
Labour intervention (*n* %)			
Induction of Labour	7,337 (36.0%)	622 (23.4%)	0.54 (0.49–0.59)
Pain management	19,274 (94.6)	2,617 (98.8)	2.08 (1.43–3.17)
Antibiotic administration	2,877 (14.1)	2,466 (93.1)	—
Blood loss cumulative volume (ml) (*n* %)			
Less than 500 (ref.)	16,182 (79.7)	1,892 (71.5)	
501–999	2,899 (14.3)	529 (20)	1.12 (1.00–1.26)
More than 1,000	1,231 (6.1)	225 (8.5)	1.29 (1.08–1.54)
Labour complications (*n* %)			
Abruption	149 (0.7)	52 (2)	1.27 (0.85–1.86)
Hypertension	278 (1.4)	54 (2)	1.46 (1.03–2.03)
Intrapartum Haemorrhage	73 (0.4)	12 (0.5)	1.02 (0.46–2.04)
Genital tract trauma (*n* %)			
No trauma (ref.)	8,511 (41.8)	1,788 (67.5)	
1st degree perineal tear	4,102 (20.1)	250 (9.4)	0.31 (0.27–0.36)
2nd Degree Perineal Tear	4,249 (20.9)	224 (8.5)	0.26 (0.23–0.31)
3rd Degree Perineal Tear	382 (1.9)	36 (1.4)	0.44 (0.31–0.63)
Episiotomy	2,812 (13.8)	345 (13)	0.5 (0.43–0.57)
Other	310 (1.5)	7 (0.3)	0.12 (0.04–0.28)
Postnatal complications (*n* %)			
Anaemia	419 (2.1)	74 (2.8)	1.11 (0.82–1.47)
Eclampsia	65 (0.3)	11 (0.4)	0.45 (0.17–1.02)
Hypertension/Pre-eclampsia	776 (3.8)	120 (4.5)	0.85 (0.65–1.1)
Retained product of conceptus	41 (0.2)	7 (0.3)	1.32 (0.47–3.12)

a CI, confidence interval; ref, reference group.

b Hospital-based midwifery care is defined as a non-continuity model of care whereby women have pregnancy visits in a hospital-based midwifery clinical and then labour and birth care with on-duty midwives.

c Threatened premature labour was defined as signs of labor before 37 weeks of gestation, without changes to the cervix or rupture of the amniotic membranes.

When comparing pregnancy characteristics between women with pyrexia, infection, or sepsis and those without, the gestational age at the first antenatal visit was comparable between the two groups (17.0 ± 6.3 vs. 17.6 ± 5.9 weeks, OR: 1.0, 95% CI: 0.99–1.01). The average number of antenatal visits was lower among women with pyrexia, infection, or sepsis compared to those without, 7.9 ± 3 vs. 8.2 ± 3 visits.

Hospital-based midwifery care (where women have pregnancy visits in midwifery clinic and then labour and birth care with on-duty midwives i.e., a non-continuity model) was the most common mode of care received. Compared to other models of care (medically-led hospital care was the reference standard) hospital-based midwifery care was associated with an increased risk of pyrexia, infection or sepsis (OR: 1.97, 95% CI: 1.54–2.56). Women with no previous births were more likely to experience pyrexia, infection, or sepsis (53.4% vs. 39.1%), with higher parity (1–4 + previous births) associated with a lower risk of pyrexia, infection or sepsis, with ORs ranging from 0.66 to 0.73. Although women with multiple pregnancies were more than twice as likely to have pyrexia, infection or sepsis (6.8% vs. 3.1%) this was not significant on the modelled odds ratio (OR: 1.02, 95% CI: 0.81–1.27). Women with pyrexia, infection or sepsis were more likely to experience threatened premature labour (OR: 1.82, 95% CI 1.46–2.27) and PPROM (OR: 4.58, 95% CI 3.77–5.56).

As expected, elective CS was performed at higher rates among women with pyrexia, infection or sepsis (13.1% vs. 12.7%, OR: 2.77, 95% CI: 2.27–3.37) and more common among women having an emergency CS (OR: 5.32, 95% CI: 4.53–6.26). Instrumental deliveries and vaginal breech were more frequent among women with pyrexia, infection or sepsis. Vaginal births without induction were less common among women with pyrexia, infection or sepsis, compared to those without (9.4% vs. 25.3%) and documented antibiotic administration was higher in women with pyrexia, infection or sepsis, compared to those without (93.1% vs. 14.1%).

Women with pyrexia, infection or sepsis had higher cumulative blood loss, with 8.5% experiencing >1,000 ml estimated blood loss compared to 6.1% among those without (OR: 1.29, 95% CI: 1.08–1.54). Intrapartum hypertension was also more common among women with pyrexia, infection, or sepsis, (OR: 1.46, 95% CI: 1.03–2.03).

### Secondary outcomes

According to SGH clinical guidelines (19), all women with prophylactic indications (except, at the time of data collection, those undergoing operative vaginal delivery) should receive antibiotic prophylaxis in alignment with WHO recommendations. However, only 34.6% of women who had a CS and 29.4% of those with 3rd or 4th degree perineal tears were documented as having received antibiotics. Among women who were GBS positive, only 63.8% were documented to receive antibiotics, and this figure dropped to 57.8% for those with PPROM. For operative vaginal deliveries, only 25.6% of women were documented as receiving antibiotics. In this subgroup of women at SGH, 76.7% of women with intrapartum pyrexia, infection or sepsis received antibiotics according to documentation (Table 2).

**Table 2:** Indication for antibiotic prophylaxis^a^.

Characteristics	Number of women	Antibiotics given *n* (%) of women with indication
Caesarean section	2,197	
Any antibiotics documented		761 (34.6)
Antibiotics documented for indication^b^		359 (16.3)
GBS positive	982	
Any antibiotics documented		627 (63.8)
Antibiotics documented for indication		568 (57.8)
PPROM	200	
Any antibiotics documented		115 (57.5)
Antibiotics documented for indication		35 (17.5)
3rd or 4th degree perineal tears	194	
Any antibiotics documented		57 (29.4)
Antibiotics documented for indication		25 (12.9)
Operative vaginal delivery	1,320	
Any antibiotics documented		338 (25.6)
Antibiotics documented for indication		—
Treatment indications		
Intrapartum pyrexia, infection or sepsis	473	363 (76.7)
Any antibiotics documented		266 (56.2)
Antibiotics documented for indication		

a Indications based on St George Hospital guidelines (except for operative vaginal delivery).

b The reason for giving antibiotics was specifically listed as this indication.

Insufficient data to assess the indication of Manual removal of the placenta.

Antibiotics given for other indications (*n* = 235).

(—) indicates no data available.

GBS screening was documented for 72.6% of cases, with 19.4% of the 5,051 swabs testing positive for GBS. Among the 982 (19.4%) positive cases, 40.0% of GBS organisms were resistant to at least one antibiotic, with a median resistance to 5 antibiotics per organism. Notably, there was no resistance to penicillin and ampicillin, but 33.4% of GBS organisms were resistant to erythromycin and 30.9% were resistant to clindamycin (Table 3).

**Table 3:** GBS Screening and data characteristics^a^.

Characteristic	No. of women
	*N* (%)
All women	6,954 (100.0)
GBS swabs sent to NSW Health Pathology	5,051 (72.6)
Positive swab	1,506 (29.8)
Swab result	
GBS	982 (19.4)
Other	524 (10.4)
Other Streptococcus species	214 (4.2)
Candida albicans	144 (2.9)
Resistant GBS organisms (*n*, % of identified GBS organisms)	393 (40.0)
Median no. of antibiotics that a GBS organism is resistant to^a^ (+ IQR)	5 (4.0–5.0)
Resistance of major antibiotics used to treat GBS infection (*n*, % of identified GBS organisms)	
Penicillin/Ampicillin	0 (0.0)
Erythromycin	328 (33.4)
Clindamycin	303 (30.9)
Other	377 (38.4)

a These data do not include swabs taken at private pathology providers outside of hospital.

b Median number of antibiotics that a single resistant GBS organism is resistant to.

Beyond GBS, other common organisms identified included Streptococcus species and Candida albicans. Women with clinical signs of infection or sepsis had a higher rate of positive GBS swabs compared to those with non-specific pyrexia or without infection.

## Discussion

This study provides insights into the prevalence and risk factors of maternal pyrexia, infection, and sepsis, as well as the use of antibiotic prophylaxis in accordance with WHO guidelines and the prevalence of antimicrobial resistance among women with GBS giving birth in different settings across NSW, Australia. A history of substance use, lower influenza vaccination rates, non-continuity midwifery care and nulliparity were associated with higher rates of pyrexia, infection and sepsis. PPROM, threatened premature labour, emergency CS and instrumental births were also associated with an increased risk of pyrexia, infection, or sepsis. It was documented that women were given antibiotic prophylaxis less than 70% of the time when indicated. Approximately 3 in 10 women screened were GBS positive, with high resistance rates to erythromycin and clindamycin observed (>30%).

Most factors associated with maternal pyrexia, infection and sepsis aligned with those found in previous studies documenting maternal sepsis risk factors. CS and instrumental birth support previous literature showing they increase the risk of maternal sepsis (4, 11, 12, 14, 15, 20, 21). The factors of no previous births, pregnancy-related hypertension, CS and postpartum haemorrhage were associated with maternal infections according to a global study of risk factors for maternal sepsis (22). Women receiving hospital-based midwifery care (a non-continuity model of care), where women have pregnancy visits in midwifery clinic and then labour and birth care with on-duty midwives were at increased risk of developing pyrexia, infection or sepsis and this is a new finding. The findings of this study strengthen the results of previous research, emphasise the clinical importance of these risk factors for maternal and neonatal morbidity and for the first time report collective risk factors across women with pyrexia, infection or sepsis. Major sepsis risk factors identified in previous studies such as age, diabetes, obesity, preeclampsia/eclampsia, multiple gestation birth and preterm birth were not significantly associated with maternal pyrexia, infection or sepsis in our study. This difference may be due to our grouping of pyrexia with infection and sepsis. We justify this grouping because: (1) previous studies have generally looked at risk factors for maternal sepsis alone, whereas we were able to demonstrate relative consistency of risk across the three combined groups contributing something novel to the literature, and; (2) as maternal temperature rises minimally during normal labor meaning all significant temperature increases likely represent a pathologic process, with a dose-dependent relationship between fever severity and increased maternal and neonatal morbidity.

According to WHO guidelines (7), maternal antibiotic prophylaxis is indicated for all women with CS, PPROM, GBS infection, 3rd or 4th degree perineal tears and manual removal of the placenta. There was insufficient detail in the data to examine the indication of manual removal of the placenta and instrumental birth. There is limited research on antibiotic prophylaxis compliance rates for indications other than GBS infection, but GBS prophylaxis has been found to be greater than 80% in both metropolitan and regional areas in NSW and Melbourne (23–25). Therefore, it is unlikely that the actual maternal antibiotic prophylaxis rates at the hospitals studied were as low as our results indicated. Instead, the low rates found are most likely due to a lack of documented evidence regarding when and why antibiotic prophylaxis was given. In the eMaternity data, the reason for antibiotic prophylaxis was not always given and answers were not standardised. There are also limitations in the available data as there may be other relevant data in other hospital records such as surgical records or in the general medical database rather than the maternity database. Additionally, during the study period fully electronic inpatient prescribing was instituted, which is not directly linked to the eMaternity record. Due to these factors, it is difficult to ascertain the true adherence to antibiotic prophylaxis guidelines from the results. This requirement to examine multiple data sources to accurately capture rates of adherence to guideline therapy is an important finding in itself, and a regrettable impediment to study of this topic.

The maternal GBS screening rate of 72.6% is comparatively lower and the positive GBS rate of 29.8% is comparatively higher than similar studies conducted in NSW and Melbourne (23, 25). These found GBS screening rates of 76%–85% and positive swab rates of 19%–22%. However, the GBS screening rate measured in this study was calculated from swabs sent to public pathology services, so the lower rate may be due to missing data from private pathology clinics. The antibiotic resistance findings of no penicillin/ampicillin resistance are reassuring as these are the first line drugs for GBS prophylaxis (26). This implies benzylpenicillin continues to be a viable and safe first-line choice for GBS prophylaxis. The high clindamycin and erythromycin resistance rates align with the Third Australian Report on Antimicrobial Use and Resistance in Human Health (AURA) 2019 (10), which found that benzylpenicillin resistance is uncommon whereas clindamycin and erythromycin resistance is around 30%. This is concerning as resistance rates for these antibiotics were respectively 4.2% and 6.4% a decade ago (8). It is important for women with a documented penicillin allergy to explore their “allergy” history and establish true allergy where possible rather than a reflex restriction of antibiotic choice. The finding that rates of resistant GBS organisms are higher in women with clinical signs of infection is of note and should be considered by staff when a GBS positive woman becomes febrile intrapartum (usually despite prophylactic antibiotics) in terms of guiding broad-spectrum antibiotic choice.

### Strengths and limitations

Strengths of this study include its multicentre nature, including a mix of metropolitan, regional and rural hospitals to identify risk factors and the comprehensive linkage of pathology and clinical datasets at one hospital, which enabled an assessment of antibiotic prophylaxis and resistance. However, there are also significant limitations. There is a high likelihood of significant issues with data accuracy and completeness due to the use of routinely collected health administrative data used to conduct this study. Inconsistencies in the dataset and incomplete recording of antibiotic prophylaxis and other relevant data may impact the accuracy of the reported findings. As a retrospective, registry-based study, misclassification or insufficient detail in the extracted data, especially for antibiotic prophylaxis indications, was also a substantial limitation. For example, some antibiotic data for women who underwent CS may have been missed as theatre and electronic prescribing records were not reviewed. There were also major inconsistencies in the eMaternity dataset with free-form responses, resulting in missing or unreliable data. Due to the limitations of the database we were unable to describe the suspected origin of pyrexia, infection or sepsis in this cohort and unable to identify how much was secondary to other infection sources, for example influenza or chorioamnionitis. We included PPROM but were unable to categorise spontaneous and indicated preterm labor due to limitations of the database. We acknowledge the limitation that these clinical syndromes overlap but the infectious risks are substantially different. Finally, though we removed antibiotic administration from the multivariable analysis we acknowledge that it may also be a confounder as it might be administered in response to fever. Fever is a side effect particularly for beta-lactam antibiotics which are commonly used in obstetric patients.

## Conclusion

Risk factors associated with the development of maternal pyrexia, infection, and sepsis include a history of substance use, low influenza vaccination rates, non-continuity midwifery care, low parity, PPROM, threatened premature labour, emergency CS, and instrumental deliveries. Documented antibiotic prophylaxis in accordance with guidelines was suboptimal, implying a requirement for improved data collection in routinely collected maternity data. Antimicrobial resistance to erythromycin and clindamycin has increased substantially, warranting further consideration in future research.

## Data Availability

The data analyzed in this study is subject to the following licenses/restrictions: Datasets can be shared upon request. Requests to access these datasets should be directed to kthompson@georgeinstute.org.
