# Supplementary material for: Risk factors for maternal pyrexia, infection and sepsis in four hospitals providing maternity care in New South Wales, Australia: a cohort study
Source: Front Glob Womens Health. 2025 Sep 26;6:1532500. doi: 10.3389/fgwh.2025.1532500 (PMC12511272; doi:10.3389/fgwh.2025.1532500)
Supplement: Supplementary file 1 [file Table1.docx]

Supplementary Material

# Supplementary Table 1

| **Demographic Characteristics** | **All women**  **n=23016** |
| --- | --- |
| Age in years (Mean ± SD) | 30.4±5.4 |
| **Body Mass Index categories (n%)** |  |
| Underweight (<18.5) | 951 (4.1) |
| Normal (18.5 – 24.9) | 10407 (45.2) |
| Overweight (25.0 – 29.9) | 5822 (25.3) |
| Obese (>30.0) | 5836 (25.4) |
| Born in Australia (n %) | 14109 (61.3) |
| **Marital Status (n %)** |  |
| Married/De-facto | 17348 (75.5) |
| Never married | 5012 (21.8) |
| Divorced | 132 (0.6) |
| Other | 485 (2.1) |
| History of endocrine disease (n %) | 3615 (15.8) |
| History of surgery (n %) | 6378 (27.8) |
| Substance use (n %) | 293 (1.3) |
| Alcohol use (n %) | 652 (2.8) |
| Smoking (n %) | 2842 (12.4) |
| History of influenza vaccination (n %) | 6418 (27.9) |
| History of pertussis vaccination (n %) | 9950 (43.2) |
| **Type of hospital (n %)** |  |
| Regional | 275 (1.2) |
| Rural | 87 (0.4) |
| Tertiary | 22653 (98.4) |
